# Antifungal and Antiochratoxigenic Activities of Essential Oils and Total Phenolic Extracts: A Comparative Study

**DOI:** 10.3390/antiox6030044

**Published:** 2017-07-09

**Authors:** Rachelle EL Khoury, Ali Atoui, Florence Mathieu, Hiba Kawtharani, Anthony EL Khoury, Richard G. Maroun, Andre EL Khoury

**Affiliations:** 1 Laboratoire de Mycologie et Sécurité Alimentaire (LMSA), Centre d’analyse et de Recherche (CAR), Campus des Sciences et Technologie, Université Saint-Joseph, Dekwaneh-Beyrouth 1104-2020, Lebanon; rachelle.khouryel@net.usj.edu.lb (R.E.K.); hiba.kawtharani@net.usj.edu.lb (H.K.); anthony.khoury4@net.usj.edu.lb (Ant.E.K.); richard.maroun@usj.edu.lb (R.G.M.); andre.khoury@usj.edu.lb (And.E.K.); 2 Laboratoire de Génie Chimique, Université de Toulouse, Centre National de Recherche Scientifique (CNRS), Institut National Polytechnique de Toulouse (INPT), Université Paul Sabatier (UPS), Toulouse 31326, France; 3 Laboratory of Microbiology, Department of Natural Sciences and Earth, Faculty of Sciences I, Lebanese University, Hadath Campus, P.O. Box 5 Beirut, Lebanon; a.atoui@cnrs.edu.lb

**Keywords:** ochratoxin A, essential oils, total phenolic extracts, biocontrol, *Aspergillus carbonarius*

## Abstract

This study is intended to prevent ochratoxin A (OTA) production by *Aspergillus carbonarius* S402 using essential oils (EOs) and total phenolic compounds extracted from plants and herbs. The EOs used in this study are the following: bay leaves, cumin, fenugreek, melissa, mint, and sage. As for the phenolic compounds, they were extracted from bay leaves, cumin, fenugreek, melissa, mint, sage, anise, chamomile, fennel, rosemary, and thyme. The experiments were conducted on Synthetic Grape Medium (SGM) medium at 28 °C for 4 days. OTA was extracted from the medium with methanol and quantified using HPLC (High Performance Liquid Chromatography). Results showed that EOs had a greater impact than the total phenolic extracts on the OTA production. Reduction levels ranged between 25% (sage) and 80% (melissa) for the EOs at 5 µL mL^−1^, and 13% (thyme) and 69% (mint) for the phenolic extracts. Although they did not affect the growth of *A. carbonarius*, total phenolic extracts and EOs were capable of partially reducing OTA production. Reduction levels depended on the nature of the plants and the concentration of the EOs. Reducing OTA with natural extracts could be a solution to prevent OTA production without altering the fungal growth, thus preserving the natural microbial balance.

## 1. Introduction

Ochratoxin A (OTA) is a mycotoxin produced by several species belonging to the *Aspergillus* and *Penicillium* genera. Its presence in common foodstuff and beverages has aroused significant public concern. More particularly, this toxin can be found in numerous food crops, such as cereals and cereal by-products [1], grapes and grape by−products [2], spices [3], and coffee [4]. Moreover, due to the physical stability of OTA, and considering the fact that mycotoxins can be transferred through the food chain, OTA can also be found in tissues and products of animal origins, such as pork and poultry, and dairy products among others [5,6]. The greatest concern about this food contaminant lies within its nephrotoxic, carcinogenic, teratogenic, and immunotoxic effects on human health following its ingestion [7,8,9]. Moreover, the International Agency for Research on Cancer has enlisted the OTA in the group 2B as a possible human carcinogen [10]. The European Union (EU), as well as many other countries, has set maximum permitted levels of this toxin on a variety of food and feed to limit the risk of human exposure [11].

OTA production and fungal growth are associated with environmental and nutritional factors such as pH, temperature, water activity, and nitrogen and carbon sources [12]. Nevertheless, little is known about the oxidative stress and antioxidants’ effects on the OTA production as well as the fungal growth of *A. carbonarius.* The correlation between the oxidative stress as well as the reactive oxygen species and mycotoxin production was firstly evaluated in 2000 by Jayashree Subramanyam [13] who found that the increasing oxidative stress induced the aflatoxins production, leading to enhanced lipid peroxydation and the generation of reactive oxygen species. While the oxidative stress induces mycotoxin production, the presence of antioxidant molecules in the culture media reduces the production of such mycotoxins (aflatoxins, fumonisins) [14,15,16]. These antioxidants can be found in plants extracts such as the essential oils (EOs) and phenolic compounds that have proven their efficiency against mycotoxin production. As for the OTA, numerous studies were conducted on the capacity of EOs to reduce fungal growth as well as the production of this particular toxin. In our previous work, we studied the effect of several EOs, such as fennel, rosemary, celery, chamomile, oregano, cinnamon, and thyme on the growth of *A. carbonarius* and OTA production. In fact, the EOs of thyme, cinnamon, and oregano were found to be effective against *A. carbonarius* [17]. On the other hand, the EOs of the fennel, chamomile, and celery were only effective against OTA production by *A. carbonarius*, suggesting a different mode of action of the studied EOs.

Nevertheless, the effect of naturally extracted total phenolic compounds on the growth of *A. carbonarius* and its OTA production has not been yet evaluated. Therefore, the aim of this study was to focus on reducing the production of OTA and establishing a comparative study between total phenolic extracts extracted from the anise, bay leaves, chamomile, cumin, fennel, fenugreek, melissa, mint, rosemary, sage, and thyme as well as their corresponding EOs. The activity of five of these EOs (anise, chamomile, fennel, rosemary, and thyme) was tested in our previous work.

## 2. Materials and Methods

### 2.1. Plant Material

In this study, the phenolic compounds were extracted from 11 plants that we purchased from a Lebanese market. The plants were the following: anise, bay leaves, chamomile, cumin, fennel, fenugreek, melissa (lemon balm), mint, rosemary, sage, and thyme.

The EOs were also purchased from a Lebanese market: mint, cumin, melissa, bay leaves, fenugreek, and sage.

### 2.2. Extraction of Phenolic Compounds from Medicinal Plants

The extraction of the phenolic compounds commenced by grinding all the 11 dried plants with a mixer until they were transformed into a fine powder. Then, 10 g of each plant powder were mixed with 100 mL acetone (85:15 v/v) and incubated for 24 h at 37 °C in a shaker/incubator (Model SI−45, serial number AS-SI45-1038E). After the maceration, the mix was then filtered through 150 mm filters (grade 290, particles retention size 80 g/m^2^, Sartorius filter discs, AG-37070, Goettingen, Germany). In order to have a filtrate free from microorganisms and fungal spores, we proceeded to perform a second filtration with 0.22 μm filters (Ministart Syringe Filter, Sartorius Stedium Biotech GmbH-37070, Goettingen, Germany). Finally, the mixture was incubated at 45 °C for 24 h to evaporate the acetone and obtain a sterile powder that was later blended with 4 mL of sterilized water. Vortexing the mixture was a necessary step to ensure its homogeneity. As for the EOs, they were bought from a Lebanese market with no further modifications.

### 2.3. Quantification of the Extracted Phenolic Compounds

In order to estimate the total phenolic contents in the previously prepared mixture, a series of gallic acid dilution is required (for the standard curve), and the results were calculated and expressed as µg GAE (Gallic Acid Equivalents). The amount of total phenolics was determined using the Folin−Ciocalteu method. In this method, 5 mL of distilled water were added to a 10 mL volumetric flask. A suitable volume of each plant extract was added to the flask along with 0.2 mL of the Folin−Ciocalteu reagent and mixed well. After 3 min of incubation in the dark, 0.4 mL of saturated Na_2_CO_3_ solution was added to the mixture, and the volume was completed with distilled water. After 1 h in the dark, the absorbance was measured with a spectrophotometer (Biotech engineering Management Co. LTD. UK, UV-9200) set at 725 nm. 

### 2.4. Strains and Culture Conditions

The *A. carbonarius* S402 strain used in this study was isolated from Lebanese grapes by El Khoury et al. [18]. The *A. carbonarius* strain S402 was sub-cultured on Czapec-Yeast-Agar medium (CYA) for 4 days at 28 °C. The CYA medium was prepared as described by El Khoury, Rizk, Lteif, Azouri, Delia, and Lebrihi [18]. After the incubation period, a spores suspension was prepared by adding 8 mL of a sterile Tween 80 (0.05%) to the surface of the culture, followed by a gentle scrapping with a sterile Pasteur Pipette (Chase Scientific Glass, Inc., Rockwood, TN, USA). The evaluation of the spores’ concentration was conducted with a Neubauer haemocytometer (Superior, Marienfield, Lauda-Konigshofen, Germany) that helped adjust the concentration to 10^6^ spores mL^−1^. The spores’ suspension was then held at 4 °C for further use. 

The effect of the extracted phenolic compounds as well as the EOs on the OTA production was evaluated on SGM medium (Synthetic Grape Medium) prepared following the instructions described by Bejaoui et al. [19].

For the phenolic compounds, an equivalent of 250 µg GAE mL^−1^ of each extract was added to 20 mL of SGM medium, followed by the addition of 1 mL of the previously prepared spore solution in the center of the Petri dishes. As for the EOs, we followed the same procedure described in our previous work [17]. In order to compare and evaluate the effect of the total phenolic compounds as well as the EO on OTA production, a control culture was prepared without adding either of the phenolic extracts or the EOs to the medium. 

All assays were carried out in triplicates for each condition. 

### 2.5. Growth, Fungal Weight Analysis, and Morphological Alteration of A. Carbonarius S402

The evaluation of *A. carbonarius* dry weight (g) as well as its radial growth (cm) were conducted by placing a sterilized transparent cellophane film on the surface of the SGM medium previously mixed with either phenolic compounds or EOs. This step was followed by the addition of 10^6^ spores mL^−1^ of the *A. carbonarius* spore solution in the center of the cellophane film. After 4 days of culture at 28 °C, *A. carbonarius* dry weight was evaluated by weighing the mycelium after its desiccation at 90 °C for 24 h. The radial growth was assessed by measuring the cultures’ diameters after the incubation period.

All assays were carried out in triplicates for each condition. 

The morphological effects of the six EOs and 11 phenolic extracts on *A. carbonarius* were evaluated macroscopically. We evaluated their impact on the color of the fungus (sporulation), thickness, and mycelium production.

### 2.6. OTA Extraction and HPLC (High Proficiency Liquid Chromatography) Analysis

The OTA was extracted from the SGM medium by removing three agar plugs (0.5 cm diameter) from each culture condition as well as the control. After placing them in 3 mL microtubes, a volume of 1 mL of HPLC grade methanol was added to the plugs. The mix was then incubated and shaken for one hour at room temperature. After centrifugation at 12,054 g, the phase containing the OTA was separated from the debris and diluted with 20 mL phosphate buffer in order to prepare the extract to be purified with immunoaffinity columns. The purification process starts by injecting the diluted OTA into Ochraprep immunoaffinity columns (R-Biopharm, Glasgow, Scotland) using a syringe. The elution of the OTA was performed by adding 1.5 mL of methanol/acetic acid (98:2, v/v) followed by 1.5 mL of distilled water. After filtering with 0.4 µm filters (Satorius stedim, Biotech, Goettingen, Germany), the OTA extract was then evaluated using a Water Alliance HPLC system equipped with an Utisphere ODB column, C18 (150 × 4.6 mm, 5 µm, 120 Å) (Interchim, Montluçon, France) at 30 °C. A 30 min isocratic flow was delivered at 49% of acidified water/acetic acid (99.8:0.2, v/v) (eluent A) and 51% of acetonitril (eluent B). The flow rate was set at 1 mL min^−1^ and the volume of the injected extract at 100 µL. The OTA was detected by a fluorescent detector set at 333/440 nm (excitation/emission wavelengths). OTA concentrations were calculated based on a standard calibration curve.

### 2.7. Statistical Analysis

All the data in this study were analyzed through one-way analysis of variance (ANOVA) and paired t-test using STATGRAPHICS Centurion XVI (version 16.1.11 for Windows, 2010, StatPoint Technologies Inc. Warrenton, VA, USA).

## 3. Results

### 3.1. Phenolic Content of the Extracts

**Figure 1:**
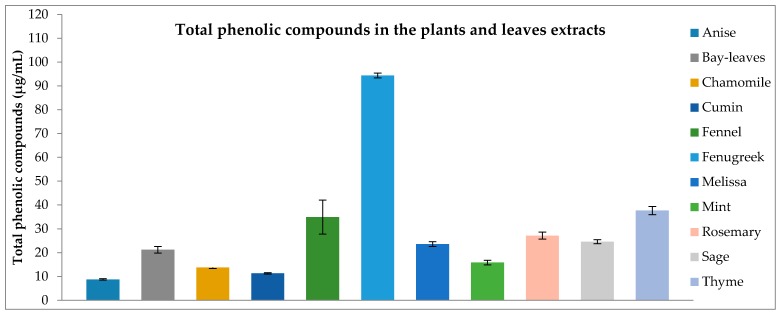
Total phenolic content of the plant and leaves extracts determined by the Folin-Ciocalteu assay and calculated as GAE in µg g^−1^ extract based on dry weight. Results are the average of triplicates ± SD (Standard Deviation). Abbreviations: GAE: Gallic acid Equivalent.

Extracts from the studied plants and leaves were quantified for their contents of phenolic compounds and expressed as GAE g^−1^ of dried plant (Gallic Acid Equivalent). The fenugreek contained the highest content of phenolics (95.21 µg GAE g^−1^ ± 1.12). The lowest phenolic contents were found in the extracts of the anise (9.05 µg GAE g^−1^ ± 0.28) and the cumin (9.62 µg GAE g^−1^ ± 3.26) as shown in Figure 1.

### 3.2. Comparison between Six Essential Oils and Phenolic Extracts on the Radial Growth of A. Carbonarius S402

The effects of the extracted phenolic compounds and the EOs on the radial growth of *A. carbonarius* are summarized in Table 1.

Our results showed that these extracts affected the radial growth, depending on the nature of the extract (Table 1). Notably, the six phenolic extracts (bay leaves, cumin, fenugreek, melissa, mint, and sage) stimulated the radial growth almost in the same manner (26.25%, 30.01%, 23.3%, 18.05%, 21.9%, and 26.7%, respectively). As for the EOs, at 1 µL mL^−1^, the bay leaves, cumin, fenugreek, and melissa also enhanced the radial growth by 20.9%, 17.25%, 18.05%, and 25.59%, respectively. At 5 µL mL^−1^, these increments were lower, and reached 18.73%, 8.24%, 10.06%, and 4.8% for the aforementioned EOs, respectively. The mint and sage EOs had an opposite effect on the radial growth, as they reduced it by 28.30% and 18.06% for the mint EO at 1 and 5 µL mL^−1^, respectively, and 44.06% and 50.84% for the sage EO at 1 and 5 µL mL^−1^ respectively.

### 3.3. Morphologic Changes in A. Carbonarius Cultured with EOs and Phenolic Compounds

**Figure 2:**
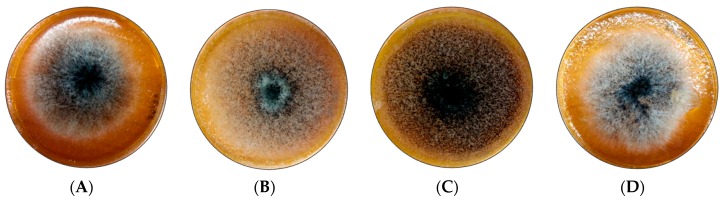
Morphological aspects of *A. carbonarius* S402 in Synthetic Grape Medium (SGM), at 28 °C for 4 days. (**A**): Control, (**B**) Cumin phenolic extracts, (**C**) Bay leaves phenolic extracts, and (**D**): mint EO at 1 µL mL^−1^.

Adding EOs or phenolic extracts to the SGM medium resulted in a change of the macroscopic morphology of all *A. carbonarius* S402 cultures (compared to the control represented in Figure 2A). Surprisingly, the extracts and EOs that enhanced the radial growth of the fungus reduced its capacity of sporulation. For example, melissa, cumin, fenugreek, sage, and mint phenolic extracts did increase the radial growth, nevertheless, the cultures seemed airy, light, and the white mycelium was dominant over the typical black spores of *A. carbonarius* (Figure 2B). The bay leaves extracts, on the other hand, increased the sporulation of *A. carbonarius*, as shown in Figure 2C. These morphological aspects were similar for the EOs: even though some EOs did not significantly affect the fungal radial growth they had a great impact on the sporulation. For example, the radial growth of *A. carbonarius* cultured with mint EO at 1 µL mL^−1^ (Figure 2D) did not change significantly, but the culture was thick, dense, and dominated by the white mycelium.

### 3.4. Comparison between Phenolic Extracts and Essential Oils on Dry Weight and OTA Production

**Figure 3:**
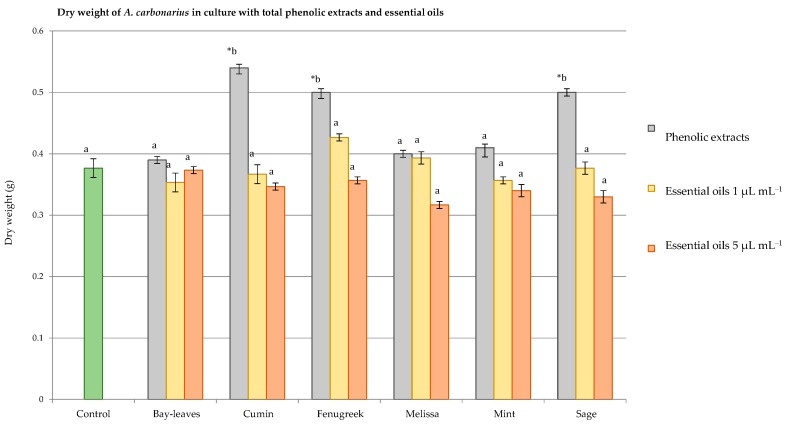
Graphical comparison between phenolic extracts and essential oils at 1 and 5 µL mL^−1^ on the dry weight of *Aspergilluscarbonarius* S402 cultured in a synthetic grape medium at 28 °C for 4 days. Statistical differences are indicated as: * = significant difference (*p* < 0.05). Data with the same letters are not significantly different (*p* < 0.05).

**Figure 4:**
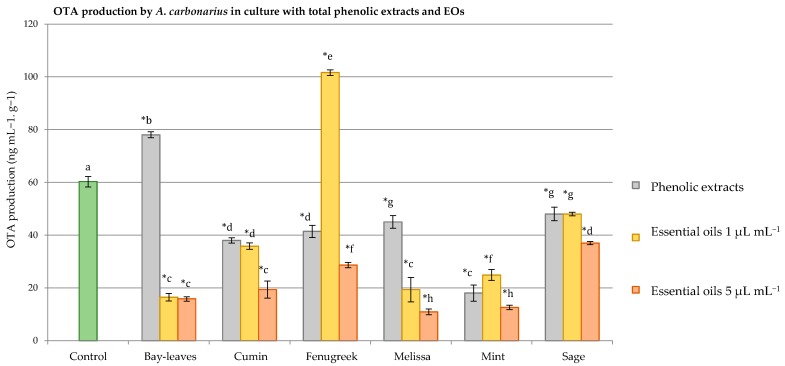
Graphical comparison between the effects of phenolic extracts and essential oils at 1 and 5 µL mL^−1^ on the ochratoxin A (OTA) produced by *A. carbonariuss* S402 cultured in a synthetic grape medium at 28 °C for 4 days. Statistical differences are indicated as: * = significant difference (*p* < 0.05). Data with the same letters are not significantly different (*p* < 0.05).

When added to SGM medium, the six EOs, showed a great impact against OTA produced by *A*. *carbonarius*, without having a pronounced effect on the fungal weight for most of the EOs. Figure 3 and Figure 4 illustrate the differences between total phenolic extracts and EOs on the fungal dry weight and OTA production.

Results showed that neither the EOs nor phenolic extracts were able to completely block the growth of *A. carbonarius* S402. In fact, none of the six EOs (bay leaves, cumin, fenugreek mint, melissa, and sage) significantly affected the dry weight of the studied fungus. On the other hand, the cumin, fenugreek, and sage phenolic extracts increased the fungal weight instead of reducing it, by 30.3% for the cumin and 22% equally for the fenugreek and sage. These weight increments were in correlation with the sporulation of *A. carbonarius* that was also increased by these extracts, whereas the phenolic extracts of the bay leaves, melissa, and mint did not significantly reduce the fungal weight of *A. carbonarius*.

Concerning the OTA production, both phenolic extracts and EOs acted differently when added to *A. carbonarius* cultures. For example, some EOs at 1 and 5 µL mL^−1^ severely affected the OTA, while the phenolic extracts had a mild impact on the production of that toxin. More particularly, cumin EO at 1 µL mL^−1^ as well as its phenolic extract reduced equally the OTA by 37.3%, while at 5 µL mL^−1^ the OTA levels reached a reduction of 69.5%. The melissa phenolic extracts reduced the OTA by 32.3%, by 78% with the EO at 1 µL mL^−1^, and 80% with the corresponding EO at 5 µL mL^−1^.

The fenugreek and bay leaves showed irregularity in this topic. In fact, adding 1 µL mL^−1^ of fenugreek EO to the SGM medium enhanced OTA production by 45.1% while at 5 µL mL^−1^ this toxin production was reduced by 60%, while the total phenolic extracts of these seeds reduced it by 32%. As for the bay leaves, their EO at 1 and 5 µL mL^−1^ equally decreased the OTA by 72%, unlike their phenolic extracts, which increased the level of this mycotoxin by 24.5%.

Surprisingly, an even reduction of OTA reaching 25% was observed when the sage phenolic extract and EO (at both concentrations) were added to the culture medium. The mint phenolic extracts and EOs acted almost in the same manner, reducing OTA levels by 69% (phenolic extracts), 60% and 70% for the EO at 1 and 5 µL mL^−1^, respectively.

As for the results of the five remaining phenolic extracts (anise, chamomile, fennel, rosemary, and thyme), their effects on radial growth, dry weight, and OTA production are represented in Table 2.

Results showed that three of the five extracts (chamomile, fennel, and thyme) significantly enhanced the radial growth of the fungus by 27.69%, 6.7%, and 11.08%, respectively, while the other two (anise and rosemary) had no significant effect on the fungal growth. As for the dry weight, anise, fennel, and thyme did not have an impact on the growth of *A. carbonarius*, chamomile increased it by 24.5%, and rosemary reduced it by 37.6%. Regarding the OTA production, the presence of the phenolic extracts reduced the production of OTA at different levels: 47.5%, 39.3%, 32.1%, and 13.9% for the thyme, anise, chamomile, and rosemary, respectively, and no change in the OTA production was observed when the fennel extracts were added to the SGM medium.

## 4. Discussion

Medicinal plants, herbs, spices, and edible and non-edible plants were always the center of attention when it came to mycotoxin reduction and/or prevention of fungal growth. Their EOs and phenolic compounds were proven to be effective against several fungal species. In this study, EOs exhibited the best antifungal and antiochratoxigenic effect in comparison to those of ethanolic phenolic extracts. In fact, none of the phenolic extracts were able to significantly reduce *A. carbonarius* dry weight, whereas only the sage at 5 µL mL^−1^ decreased it by 10.8%. Nevertheless, phenolic extracts as well as EOs exhibited strong effects on OTA reduction. For instance, the mint was the most effective among the 11 tested phenolic extracts. It reduced OTA production by up to 70% without affecting the fungal dry weight. These results are similar to those obtained by Panda et al. [20] who found that the phenolic extracts from *Mentha arvensis*, commonly known as mint leaves, highly reduced the citrinin production by *P. citrinum* without truly affecting its growth. The use of the mint EO at 0.5 µL mL^−1^ allowed the growth of *A. ochraceus* with no detectable OTA in the culture medium [21]. In a study by Hitokoto et al. [22], the use of thyme caused a 10% to 90% growth reduction in *A. flavus*, *A. versicolor*, and *A. ochraceus* but showed complete and nonlinear inhibition of their toxin production (86% to 100%). In their study, basil leaves and cumin also partially reduced *A. ochraceus* growth but blocked its OTA production. These results are similar to ours, where these extracts acted in the same manner: the phenolic extracts of the basil leaves and the cumin as well as their EOs did not act in a proportionate way regarding fungal weight reduction and the OTA production. In fact, the phenolic extracts of the bay leaves had no significant effect on *A. carbonarius* growth but reduced OTA by 26.5%.

As for the phenolic extracts of the anise, chamomile, fennel, rosemary, and thyme, their effects on OTA production were lower than those found with the EOs in our previous study [17]. In fact, the thyme phenolic extracts reduced OTA production by 47.5% but had no effect on the growth of *A. carbonarius*. The EO of the thyme acted differently: at 5 µL mL^−1^ it completely blocked the fungal growth, and at 1 µL mL^−1^ it reduced OTA by up to 90% (this reduction was in correlation with growth reduction). As for the anise, chamomile, and rosemary, their capacity on reducing OTA production was lower than their corresponding EOs: anise phenolic extracts only reduced OTA by 35.25% although its EO reduced it by 76.6% and 83.9% (at 1 and 5 µL mL^−1^, respectively). Chamomile phenolic extracts reduced it by 32.16%, its EO at 1 µL mL^−1^ by 67.5%, and at 5 µL mL^−1^ by 78.1%. The same reduction profile was obtained with the phenolic extracts and EOs of the rosemary: the OTA was reduced by 14.01% with the phenolic extracts, 53.7% and 78.3% with rosemary EO when used at 1 and 5 µL mL^−1^, respectively. Surprisingly, the fennel phenolics did not reduce OTA although its EO at 5 µL mL^−1^ reduced it by 88.9% (the highest reduction obtained in our previous study). These differences may be attributed to the role of the active ingredients of the EOs that are implicated in the repression of the OTA biosynthetic genes expression of *A. carbonarius* (reductions reached 99.2% for the *acpks* gene responsible for the early steps of OTA biosynthesis).

Despite the rarity of studies on the antifungal and antimycotoxigenic effects of total phenolic extract, the active components found in these extracts were thoroughly studied. In general, eugenol (basil), vanillic acid (sage), caffeic acid (mint), rosmarinic acid (mint), *p-*coumaric acid (cumin), and cinnamic derivatives (bay leaves) are the main active component responsible for the antioxidant activity of the phenolic extracts [23,24,25,26,27]. The caffeic, cinnamic, ferulic and vanillic acids were proven to control aflatoxigenic fungi and AFB1 production as well as fumonisin on maize during storage [28,29]. Likewise, Palumbo et al. [30] found that OTA can be reduced by the use of different phenolic antioxidant such as 4-hydroxybezoic acid on *A. carbonarius*, *A. sulphureus*, *A. alliaceus,* and *A. lanosus*, vanillic acid on *A. albertensis* and *A. elegans*, as well as catechin on *A. sulphureus*.

Some authors reported that the antioxidant activity of different plant extracts depends on their phenolic content, while others found no such correlation, because of the presence of other active ingredients in each plant [31,32,33]. Moreover, da Cruz Cabral, et al. [34] stated that the potential of plant extracts to decrease fungal growth and/or mycotoxin production depends on their composition and the nature of the solvent used for the extraction. In fact, the solvents with a polar nature have a better penetration capacity than none polar ones, enabling them to extract a wider variety of compounds from the plant tissues. The acetone that we used in this study, as well as the methanol was found to be the best solvents for the extraction of antifungal compounds [35,36]. Furthermore, the type of the phenolic structure was proven to be more important than the concentration: molecules with a hydroxyl group having a system of delocalized electrons in the phenolic structure have the highest antioxidant activity [37]. To date, the mechanisms of action of active compounds in plant extracts are not completely understood. Nevertheless, numerous authors support the idea of the existence of three aspects for their inhibitory functions: first, the enzymes responsible for intracellular functions can be altered by the presence of −OH groups, and are able to form hydrogen bonds with those enzymes. Secondly, the rigidity and integrity of the hypha cell wall can be lost due to the interaction of these compounds with the membrane enzymes of the fungi. Lastly, the rupture of the cytoplasmic membrane can be achieved by the change of permeability of the cell membranes and granulation of the cytoplasm. In addition, the hydrophobicity of certain compounds found in the EOs extracts allows them to cross the cell membrane and change their permeability for cations such as H^+^ and K^+^, thus changing the flow of protons and the pH of the cells, affecting their chemical compositions and activities [34]. Phenolic compounds are also capable of altering the fungal cell permeability, resulting in the loss of macromolecules from the cell interior and interacting with the proteins of the membrane, leading to their structure and functionality deformation [38]. 

As for the phenolic extracts and EOs that enhanced OTA production, such as bay leaves extracts and fenugreek EO at 1 µL mL^−1^, Prakash et al. [39] reported that the use of low concentrations of *Piper betle L*. EO on *A. flavus* cultures increased AFB1 production, implicating that low fungicide doses create some stress condition which was responsible for the production of more secondary metabolites as a defense mechanism by the fungus. The same observation was noted by Wu et al. [40] who found that *F. oxysporum* biomass was reduced with ferulic acid that increased its mycotoxin production by 227.7%.

The different macroscopic aspects of *A. carbonarius* S402 between the control and the treated samples were highly noticed in this study. In fact, the extracts enhanced radial growth, and reduced the sporulation of the fungus, resulting in OTA reduction. For example, adding 250 µL mL^−1^ of cumin phenolic extracts to SGM medium caused a radial growth increment (47.8%), OTA reduction (37.3%), and the cultures were dominated by the presence of white mycelium. These transformations were observed by Ferreira et al. [41] who found that the use of curcumin EO at 1% on *A. flavus* cultures did not only reduce sporulation by 95.78%, but also decreased the viability of the remaining spores to 100%. *A. parasiticus* manifested the same results when it was cultured with thyme’s EO. In fact, after 5 days of culture, its sporulation capacity was reduced by 95% [42].

## 5. Conclusions

The natural chemical structure of phenolic compounds and essential oils provides them with the potential of being acceptable antifungal and antimycotoxigenic agents for application on food matrices. This study demonstrated that EOs could strongly reduce OTA rather than reducing the growth of *A. carbonarius*. They are more efficient than the phenolic compounds extracted from the same plants, reaching 70% and 80% for the EOs of the melissa and mint, respectively. As for the total phenolic extracts such as mint, anise, and thyme, they were capable of only partially reducing OTA production (69%, 35.25%, and 47.5%, respectively). This study is a first step toward reducing the occurrence of OTA and its daily ingestion by humans. Nevertheless, in order for it to succeed, a deeper more detailed study should be conducted on the efficiency of these natural compounds on other ochratoxigenic and mycotoxigenic species.

## Figures and Tables

**Table 1:** Radial growth (cm) of *Aspergillus carbonarius* S402 after four days of culture at 28 °C on Synthetic Grape Medium (SGM) supplemented with the phenolic extracts at 250 µg GAE mL^−1^ as well as essential oils (EOs) at 1 and 5 µL mL^−1^ each, compared to a control.

Plant Material	Radial Growth (cm)		
	Phenolic extracts	EOs at 1 µL mL^−1^	EOs at 5 µL mL^−1^
Control	5.90 ± 0.43 ^a^	5.90 ± 0.43 ^a^	5.90 ± 0.43 ^a^
Bayleaves	8.00 ± 0.34 *^,d^	7.46 ± 0.30 *^,b^	7.26 ± 0.15 *^,b^
Cumin	8.43 ± 0.47 *^,e^	7.13 ± 0.05 *^,c^	6.43 ± 0.35 *^,c^
Fenugreek	7.70 ± 1.04 *^,c^	7.20 ± 0.20 *^,c^	6.60 ± 0.10 *^,c^
Melissa (Lemon balm)	7.20 ± 0.10 *^,b^	7.93 ± 0.15 *^,d^	6.20 ± 0.20 *^,d^
Mint	7.56 ± 0.20 *^,c^	4.23 ± 0.05 *^,e^	4.80 ± 0.10 *^,e^
Sage	8.06 ± 0.15 *^,d^	3.30 ± 0.10 *^,f^	2.90 ± 0.10 *^,f^

The means of the radial growth (cm) ± the standard deviation of the triplicates are represented in this table. Statistical differences are indicated as: * = significant difference (*p* < 0.05). Data with the same letters are not significantly different (*p* < 0.05). Abbreviations: GAE: Gallic acid Equivalent, EOs: Essential oils.

**Table 2:** Radial growth (cm), dry weight of *Aspergillus carbonarius* S402, and OTA production (ng mL^−1^ g^−1^ of dry weight) after four days of culture at 28 °C on Synthetic Grape Medium (SGM) supplemented with the five phenolic extracts at 250 µg GAE mL^−1^.

Plant Material	Radial Growth (cm)	Dry Weight (g)	OTA Production (ng mL^−1^ g^−1^ of Dry Weight)
Control	5.90 ± 0.43 ^a^	0.385 ± 0.01 ^a^	60.37 ± 0.46 ^a^
Fennel	6.33 ± 0.20 *^,b^	0.486 ± 0.02 ^a^	60.25 ± 0.08 ^a^
Rosemary	5.63 ± 0.20 ^a^	0.240 ± 0.01 *^,c^	51.92 ± 1.46 *^,d^
Chamomile	8.16 ± 0.15 *^,c^	0.510 ± 0.13 *^,b^	40.95 ± 2.55 *^,b^
Anise	5.60 ± 0.30 ^a^	0.435 ± 0.05 ^a^	36.62 ± 0.54 *^,b^
Thyme	7.93 ± 0.05 *^,d^	0.433 ± 0.01 ^a^	31.64 ± 1.53 *^,c^

The means of the radial growth (cm), the dry weight (g), and OTA production (ng mL^−1^ g^−1^ of dry weight) ± the standard deviation of the triplicates are represented in this table. Statistical differences are indicated as: * = significant difference (*p* < 0.05). Data with the same letters are not significantly different (*p* < 0.05). Abbreviations: OTA: ochratoxin A, GAE: Gallic acid Equivalent.
